# Energy Metabolites as Biomarkers in Ischemic and Dilated Cardiomyopathy

**DOI:** 10.3390/ijms22041999

**Published:** 2021-02-18

**Authors:** Jan Haas, Karen S. Frese, Farbod Sedaghat-Hamedani, Elham Kayvanpour, Rewati Tappu, Rouven Nietsch, Oguz Firat Tugrul, Michael Wisdom, Carsten Dietrich, Ali Amr, Tanja Weis, Torsten Niederdränk, Michael P. Murphy, Thomas Krieg, Marcus Dörr, Uwe Völker, Jens Fielitz, Norbert Frey, Stephan B. Felix, Andreas Keller, Hugo A. Katus, Benjamin Meder

**Affiliations:** 1 Department of Internal Medicine III, University of Heidelberg, 69120 Heidelberg, Germany; jan.haas@med.uni-heidelberg.de (J.H.); karen.frese@med.uni-heidelberg.de (K.S.F.); Farbod.Sedaghat-Hamedani@med.uni-heidelberg.de (F.S.-H.); Elham.Kayvanpour@med.uni-heidelberg.de (E.K.); Rewati.Tappu@med.uni-heidelberg.de (R.T.); Rouven.Nietsch@med.uni-heidelberg.de (R.N.); OguzFirat.Tugrul@med.uni-heidelberg.de (O.F.T.); michaelwisdom03@gmail.com (M.W.); Ali.Amr@med.uni-heidelberg.de (A.A.); Tanja.Weis@med.uni-heidelberg.de (T.W.); Norbert.Frey@med.uni-heidelberg.de (N.F.); Hugo.Katus@med.uni-heidelberg.de (H.A.K.); 2 DZHK (German Centre for Cardiovascular Research), 17475 Greifswald, Germany; mdoerr@uni-greifswald.de (M.D.); voelker@uni-greifswald.de (U.V.); jens.fielitz@uni-greifswald.de (J.F.); Stephan.Felix@med.uni-greifswald.de (S.B.F.); 3 Siemens Healthcare GmbH, 91058 Erlangen, Germany; dietrich.carsten@siemens-healthineers.com (C.D.); torsten.niederdraenk@siemens-healthineers.com (T.N.); 4 MRC Mitochondrial Biology Unit, University of Cambridge, Cambridge CB2 0XY, UK; mpm@mrc-mbu.cam.ac.uk; 5 Department of Medicine, University of Cambridge, Addenbrooke’s Hospital, Hills Road, Cambridge CB2 0QQ, UK; tk382@medschl.cam.ac.uk; 6 Department of Internal Medicine B, University Medicine Greifswald, 17475 Greifswald, Germany; 7 Department of Functional Genomics, Interfaculty Institute for Genetics and Functional Genomics, University Medicine Greifswald, 17475 Greifswald, Germany; 8 Department of Bioinformatics, University of Saarland, 66123 Saarbrücken, Germany; andreas.keller@ccb.uni-saarland.de; 9 Genome Technology Center, Stanford University, Stanford, CA 94304, USA

**Keywords:** cardiomyopathy, energy metabolism, heart failure, multi-omics

## Abstract

With more than 25 million people affected, heart failure (HF) is a global threat. As energy production pathways are known to play a pivotal role in HF, we sought here to identify key metabolic changes in ischemic- and non-ischemic HF by using a multi-OMICS approach. Serum metabolites and mRNAseq and epigenetic DNA methylation profiles were analyzed from blood and left ventricular heart biopsy specimens of the same individuals. In total we collected serum from *n* = 82 patients with Dilated Cardiomyopathy (DCM) and *n* = 51 controls in the screening stage. We identified several metabolites involved in glycolysis and citric acid cycle to be elevated up to 5.7-fold in DCM (*p* = 1.7 × 10^−6^). Interestingly, cardiac mRNA and epigenetic changes of genes encoding rate-limiting enzymes of these pathways could also be found and validated in our second stage of metabolite assessment in *n* = 52 DCM, *n* = 39 ischemic HF and *n* = 57 controls. In conclusion, we identified a new set of metabolomic biomarkers for HF. We were able to identify underlying biological cascades that potentially represent suitable intervention targets.

## 1. Introduction

Heart failure (HF) is considered as a global pandemic, since it affects around 25 million people worldwide [1,2]. It is a complex syndrome with reduced ability of the heart to pump sufficient volumes of blood [3,4,5] required to meet the metabolic demands of our organ systems [4]. The concept, that heart failure is comparable to an “engine out of fuel” has been developed and decades of studies on myocardial energetics have confirmed and refined this concept [6]. Briefly, the cardiac energy metabolism has three pillars: (i) uptake and utilization of fatty acids and glucose by beta-oxidation and glycolysis, respectively, (ii) oxidative phosphorylation in mitochondria, to generate energy and (iii) energy transfer and consumption [6]. The healthy heart is a “metabolic omnivore” with flexibility in substrate consumption. Whereas under normal conditions ~60–90% of ATP is produced via fatty acid (FA) oxidation and only ~10–40% of the ATP results from pyruvate oxidation, this balance can dramatically shift [1]. Under exercise conditions, less FA but glucose is used for ATP generation. Furthermore, stressors like prolonged fasting or poorly controlled diabetes can lead to a major shift in energy production through breakdown of ketone bodies and amino acids [1]. The numerous studies that underlie our knowledge on HF-related metabolic changes used mainly mouse models [1,2].

Human clinical studies on the metabolic changes in HF are sparse. The rationale of this study was to establish key metabolic changes during HF and find common markers of the two major forms of systolic HF, namely ischemic- and non-ischemic HF. Here, we show that several energy metabolism-related markers in the serum and the mRNA levels of their rate-limiting enzymes in the heart and whole blood are altered in patients with chronic HF, rendering them diagnostic and potential therapeutic targets.

## 2. Results

### 2.1. Detection of Dysregulated Energy Metabolites

Our study used an initial stage with a multi-omics design in non-ischemic HF and a succeeding independent validation in non-ischemic as well as ischemic HF. We collected serum from *n* = 82 HF patients with DCM (mean NYHA class 2.2) and *n* = 51 clinical controls without signs or symptoms of HF (NYHA class 0) in the first stage and from *n* = 52 DCM, *n* = 39 ICM and *n* = 57 controls in the second stage. Clinical baseline characteristics for the subjects are summarized in Table 1 and Table 2.

**Figure 1:**
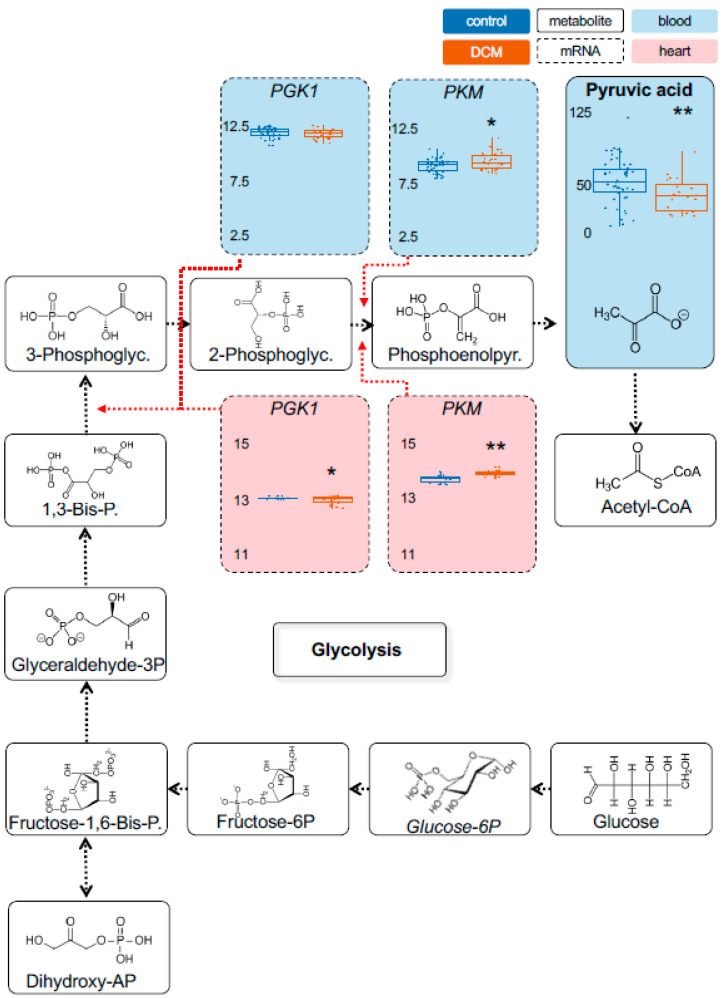
Detection of metabolic changes in glycolysis in heart failure (HF) patients. Control (CTRL) samples are depicted in blue. Values of the Dilated Cardiomyopathy (DCM) cohort are colored orange. Significance values are indicated. Boxes with dashed lines indicate mRNA expression and with full lines metabolite levels in serum (light blue shading) and left-ventricular heart tissue (pale red shading). As shown, *PKM* expression is upregulated in blood cells and myocardial tissue, while the resulting metabolite pyruvic acid was not significantly altered. Metabolite concentrations are shown as [µM]. RNAseq expression is given as (rlog-)normalized read counts. *: *p* < 0.05; **: *p* < 0.01. PGK1 = phosphoglycerate kinase 1; PKM = pyruvate kinase.

**Figure 2:**
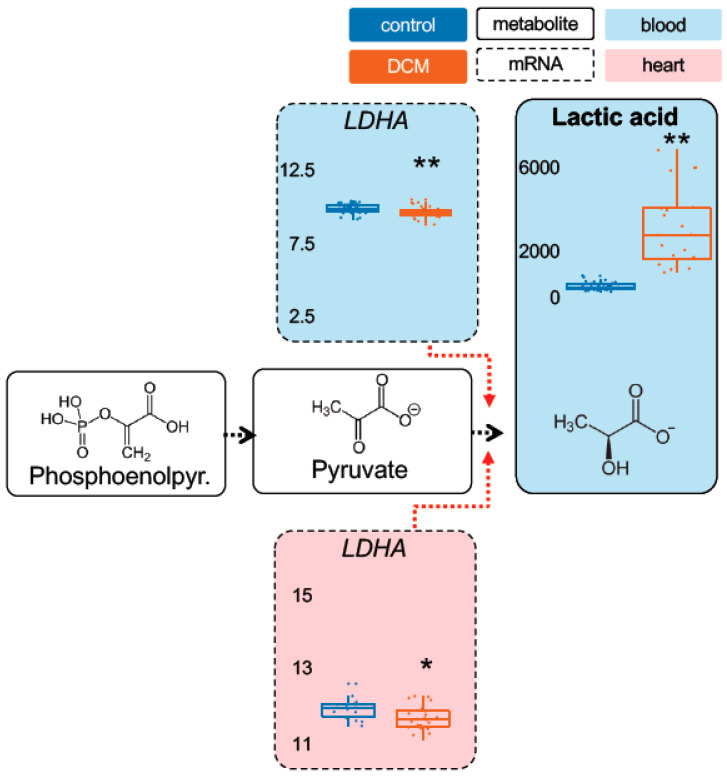
Detection of increased lactate levels. Following glycolysis, lactic acid is significantly increased in the stage 1 cohort, coinciding with decreased LDHA expression in blood but not in myocardial tissue. Same color and symbol annotation as in Figure 1. RNAseq expression is given as (rlog-)normalized read counts. Metabolite concentrations are shown as [µM]. *: *p* < 0.05; **: *p* < 0.01. LDHA = lactate dehydrogenase A.

**Figure 3:**
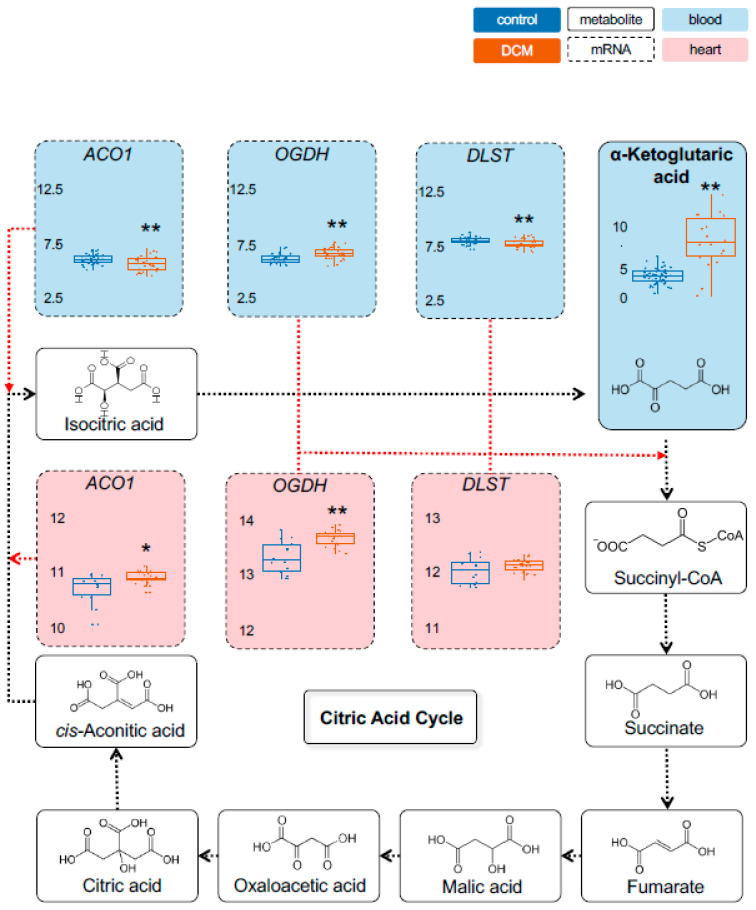
Detection of metabolic dysregulation in citric acid cycle. In the citric acid cycle, several mRNA expression changes were observed, with OGDH being upregulated in mononuclear blood cells and myocardial tissue. The metabolite α-Ketoglutaric acid was (as a consequence) strongly increased and could potentially serve as new biomarker in HF. Control (CTRL) samples are depicted in blue. Values for samples from the Dilated Cardiomyopathy (DCM) cohort are orange. Significance values are given. Boxes with dashed lines indicate mRNA and with full lines metabolomics expression in blood (light blue shading) and left-ventricular heart tissue (pale red shading). RNAseq expression is given as (rlog-)normalized read counts. Metabolite concentrations are shown as [µM]. *: *p* < 0.05; **: *p* < 0.01. ACO1 = Aconitase 1; OGDH = oxoglutarate dehydrogenase; DLST = dihydrolipoamide s-succinyltransferase.

**Figure 4:**
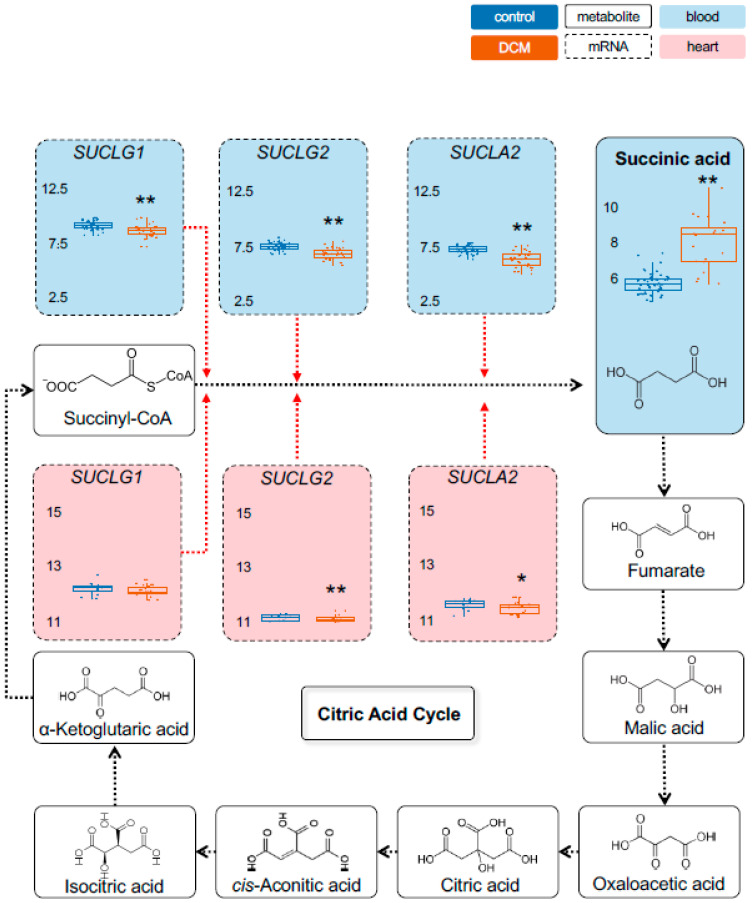
Detection of metabolic dysregulation in citric acid cycle. Following the citric acid cycle, succinic acid was Scheme 1. HF cohort, coinciding with decreased mRNA levels of several enzymes in blood. Same color and symbol annotation as in Figure 3. RNAseq expression is given as (rlog-)normalized read counts. Metabolite concentrations are shown as [µM]. *: *p* < 0.05; **: *p* < 0.01. SUCLG1 = Succinate-CoA Ligase Alpha Subunit; SUCLG2 = succinate-coA ligase GDP-forming beta subunit; SUCLA2 = succinate-coA ligase ADP-forming beta subunit.

Metabolomic measurements in the stage 1 cohorts were performed using Biocrates Metabolite assays for most important energy metabolites. While some metabolites showed unaltered levels in DCM compared to controls, such as *pyruvic acid (+oxaloacetic acid)* (Figure 1), a range of other energy metabolites were significantly changed in their serum levels. As shown by principal component analysis, cases and controls separate very well based on their metabolic profile (Supplemental Figure S2). In case of *lactate*, DCM patients showed as much as a 5.7-fold higher level compared to controls (*p* = 1.7 × 10^−6^) (Figure 2). Also, *Alpha-ketoglutaric acid*, (*p* = 4.1 × 10^−5^; 1.9-fold) (Figure 3) and *succinic acid* (*p* = 7.7 × 10^−7^; 1.4-fold) (Figure 4) showed significantly increased levels in DCM patients. Together, this data points towards a changed energy metabolism in non-ischemic HF.

### 2.2. Myocardial Epigenetic Programs and mRNA Levels of Rate-Limiting Enzymes Are Associated with HF Metabolites

To explore whether the metabolic state-change is due to expression changes in key enzymes of the detected metabolites, we compared the metabolite data to whole-transcriptome RNA-sequencing data from blood and heart muscle tissue from the same cohort of DCM patients and compared them to controls.

As proxies for the metabolites we chose the rate-limiting enzymes hexokinase 1 (HK1), muscle phosphofructokinase (PFKM), phosphoglycerate kinase 1 (PGK1) and muscle pyruvate kinase (PKM) for pyruvic acid (glycolysis). As part of the citric acid cycle we used oxoglutarate dehydrogenase (OGDH) and dihydrolipoamide s-succinyltransferase (DLST) for alpha-ketoglutaric acid, succinate-coA ligase alpha subunit (SUCLG1), succinate-coA ligase GDP-forming beta subunit (SUCLG2) and succinate-coA ligase ADP-forming beta subunit (SUCLA2) for succinic acid.

As shown in Figure 1, we found a significant increase in *PKM* (blood and heart) and a decrease in *PGK1* expression (heart) in DCM patients. We also observed a significantly increased *HK1* (blood and heart) (Supplemental Figure S3) and decreased *LDHA* (blood and heart) (Figure 2) expression. Furthermore, a significant regulation was detected for *DLST* (blood), *OGDH* (blood and heart) and *ACO1* (blood and heart) expression (Figure 3). Finally, we found a decreased expression for *SUCLA2* and *SUCLG2* (both blood and heart) and for *SUCLG1* (blood) (Figure 4). Supplemental Figure S4 is giving a comprehensive overview on the described gene expression and its validation. Of note, the corresponding methylation sites were also significantly different for those genes (Supplemental Figure S5).

### 2.3. Validation of Metabolic Dysregulation for the Development of New Biomarkers

To substantiate these initial findings on energy metabolic state-changes and to screen for robust data for the development of new biomarkers for DCM and systolic HF in general, we assessed the metabolites of the stage 1 cohorts and additional energy metabolites in independent HF cohorts (DCM, ICM, controls).

**Figure 5:**
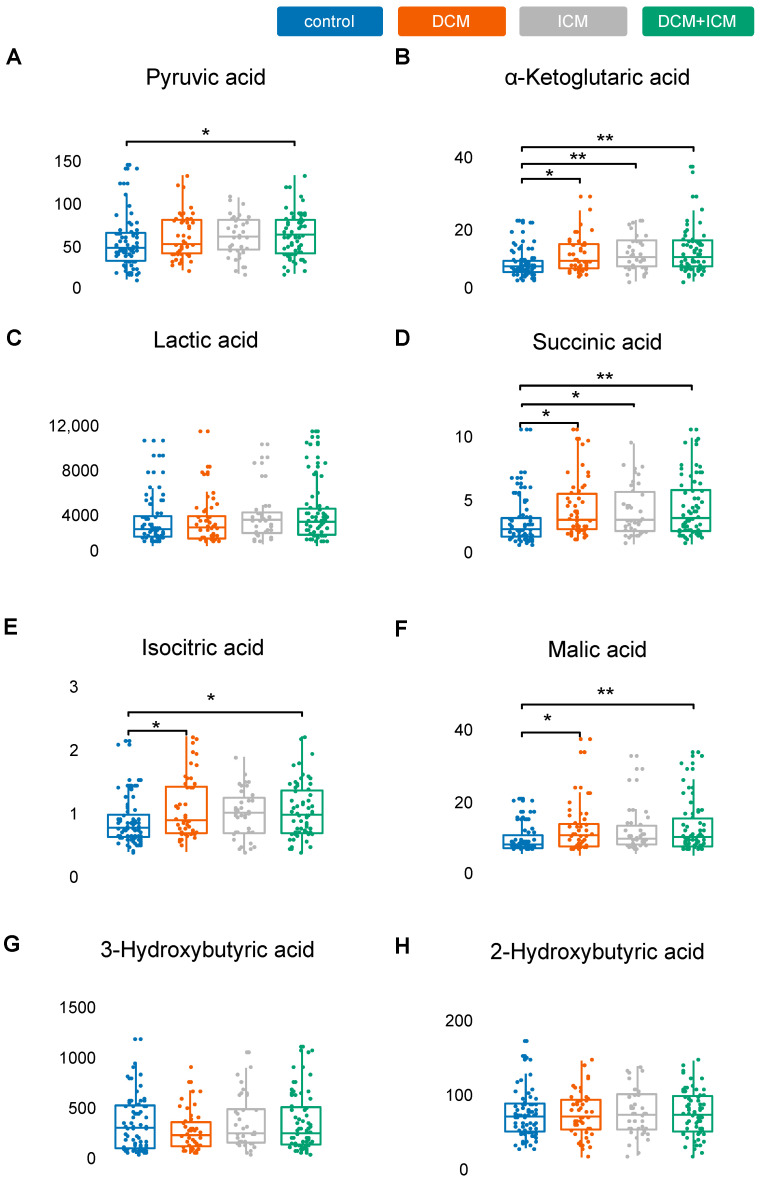
Validation in independent HF cohorts. While changes in pyruvic acid levels could not been replicated (**A**), the HF-related upregulation of α-Ketoglutaric acid (**B**) was validated, while lactic acid (**C**) levels are not significantly altered in this cohort, in contrast to succinic acid (**D**), which was successfully validated. Isocitric and malic acid were also increased (**E**,**F**). Levels for 2-Hydroxybutyric and 2-Hydroxybutyric acid were found to be unaltered (**G**,**H**). Control (CTRL) samples are depicted in blue. Values for samples from the Dilated Cardiomyopathy (DCM) cohort are colored orange. Ischemic cardiomyopathy subgroup (ICM) is painted grey. Values for the combined cohort of DCM and ICM samples (DCM/ICM) are shown in green. Significance values are given. Metabolite concentrations are shown as [µM]. *: *p* < 0.05; **: *p* < 0.01.

As shown in Figure 5, *alpha-ketoglutaric acid* and *succinic acid* (see Figure 5) were significantly increased in DCM and ICM as observed in the stage 1 cohorts.

The metabolic markers also included *pyruvic acid (+oxaloacetic acid)*, which displayed a tendency to be elevated in DCM and ICM patients but did not reach statistical significance in the subgroups. However, the combined HF cohort showed significantly elevated *pyruvic acid* levels (1.5-fold, *p* = 0.032) (Figure 5A). For *lactic acid*, our measurements showed a trend of higher levels in DCM as observed in the screening cohort but did not reach significance (Figure 5C). Additional metabolite information could be generated to fine map the energy metabolism changes. For instance, higher levels of *isocitric acid* were detected in DCM (*p* = 0.012) and in ICM (*p* = 0.067) (E). As shown in Figure 3, the rate-limiting enzyme *aconitase 1* (*ACO1*) was also significantly dysregulated in blood and heart in the multi-omics cohorts. Similarly, *malic acid* was increased in HF (DCM *p* = 0.019; ICM *p* = 0.07) (Figure 5F). For *3-hydroxybutyric acid* (Figure 5G), DCM and ICM patients had a trend to lower levels compared to controls. No difference was seen for *2-hydroxybutyric acid* (Figure 5H).

## 3. Discussion

The American Heart Association recently highlighted the potential for assessing metabolic states in cardiovascular diseases and made recommendations on future directions of human centered research in this area [7]. This is partly due to the fact that most seminal metabolic studies have been performed in vitro or in mice [8]. Although leading to important discoveries such as branched-chain amino acids (BCAA) accumulation [8], decreased cardiac fatty acid oxidation and increased ketone oxidation [9] and many other key-changes during HF and cardiac remodeling [10,11], the studies resulted in relatively little understanding of the human system and its clinical relevance as well as the underlying regulatory principles in heart disease. To the best of our knowledge, this is the first multi-OMICS study on energy metabolism in HF, combining transcriptomics and epigenomics, with quantitative metabolomics being the closest entity to the actual phenotype [12]. Since energetic changes are thought to be hallmarks of cardiac remodeling [13,14], for example, by prolonged reliance on glucose utilization rather than fatty acid oxidation, we distinctly focused on energy metabolism in this study [10].

Lactate was one of the first biomarkers for HF and we can reproduce elevated levels only in part of our HF cohorts [2]. This might be due to the differences in disease severity (although LVEF was comparable) and underlines the limitations of this marker in daily clinical practice. Other findings seem to be more robust, such as the detection of increased levels of succinic acid. As Chouchani et al. showed in an ischemia reperfusion mouse model, the selective accumulation of the citric acid cycle intermediate succinate is a universal metabolic signature of ischemia in a range of tissues including the heart [15]. They pinpointed that increased succinate levels arise from reduced conversion to fumarate by the reversal of succinate dehydrogenase (SDH), when fumarate, aspartate and malate were available [15]. We here show significantly elevated succinate and malate levels in human systolic HF, with potential therapeutic implications. The described mechanism has also been validated by Zhang et al. [16]. However, they also proposed an accumulation of succinate in ischemia predominantly via the canonical Krebs-Cycle for example, through elevated α-Ketoglutaric acid levels, which we also find in our study.

Interestingly, the metabolic changes also are reflected on the epigenetic and transcript level of several rate-limiting enzymes of this pathway, indicating that metabolic changes are at least partly driven by transcriptional regulation of the respective pathway.

Besides the biological validation in independent cohorts, we used gas chromatography mass spectrometry(GC-MS) instead of Liquid chromatography–mass spectrometry (LC-MS/MS) for robust and reproducible independent metabolite separation and quantitation in the validation phase [10]. The applicability of both methods has been recently shown for example, by Mueller et al. in a study on metabolites in a cardiac hypertrophy and heart failure model [17]. Potential limitations of this study include its monocentric nature with potential referral bias of our tertiary center. To address this, the findings have been carefully validated in three independent cohorts of HF, also including ischemic HF to show generalizability. Another potential limitation is the higher number of diabetic patients compared to controls, which could lead to a metabolic effect driven by insulin resistance [18], which is a known effect especially in diabetic and obese patients [19]. However, by adjusting our cohort to glucose (H1) levels (see M&M and Supplemental Figure S1), we prevent such a potential effect, as H1 has been shown to be significantly different in type-2 diabetes compared to controls [20].

In summary, we have detected significant dysregulation related to energy production pathways in HF at different OMICs-levels, showing its importance in disease onset and progression and suggest a possible use of distinct molecules like succinic acid as an (early) biomarker and interventional target in HF.

## 4. Materials and Methods

### 4.1. Patient Enrolment and Study Design

Our study was approved by the ethics committee of Heidelberg University. All participants have given written informed consent to allow for molecular analysis of blood and left-over tissue for the identification of novel biomarkers (appl. no. S-390/2011). The diagnosis of Dilated Cardiomyopathy (DCM) was confirmed after excluding coronary artery disease as determined by coronary angiography, valvular heart disease was excluded by cardiac magnetic resonance imaging (cMRI) and echocardiography and myocarditis/inflammatory DCM by histopathology [21]. Patients routinely biopsied after heart transplantation served as controls for epigenetic and transcriptomic studies. Importantly, only patients with normal cardiac function and without transplant rejection were included as controls.

We performed a two-step approach and recruited first a screening cohort including *n* = 82 DCM patients and *n* = 51 controls and afterwards a second stage cohort of *n* = 52 DCM, *n* = 39 ischemic cardiomyopathy (ICM) patients and *n* = 57 controls. The ICM patients in the second stage had relevant coronary artery disease as judged by coronary angiography in combination with decreased systolic function. Clinical controls were recruited at the study side and they had a cardiovascular risk profile but neither HF or relevant coronary artery disease, as judged by a recent coronary angiography.

### 4.2. Biomaterial Processing

For all subjects, blood draw was performed without requiring a special fasting protocol. For serum samples, an 8 mL aliquot of blood samples from each participant was collected directly into serum collection tubes (Sarstedt Monovette). The serum samples were allowed to stand for 1 h at room temperature before being centrifuged at 3000 rpm for 10 min. Then, the serum supernatant was recovered and stored at −80 °C until further analysis. In total 200 µL serum were sent to Biocrates (Innsbruck) on dry ice, where metabolomic profiling was performed. Biopsies for transcriptome analysis have been handled as described in [22].

### 4.3. Metabolomics Profiling

In the screening cohort the AbsoluteIDQ p180 kit, as well as Specialized assay kits including assays for Amino acids & biogenic amines, Acylcarnitines, Lysophophosphatidylcholine, Phosphatidylcholine, Sphingomyelins, Hexoses, Prostaglandins, Bile acids and the Energy metabolism (Biocrates Life Sciences AG, Innsbruck, Austria) were used to quantify in total 435 metabolites, of which 381 had detection levels meeting quality standards. Hexoses were measured using a fully automated assay based on PITC (phenylisothiocyanate)-derivatization in the presence of internal standards followed by flow injection analysis (FIA)- tandem mass spectrometry (MS/MS) using an AB SCIEX 4000 QTrap^®^ mass spectrometer (AB SCIEX, Darmstadt, Germany) with electrospray ionization. The experimental metabolomics measurement technique is described in detail by patent US 2007/0004044 accessible online at http://www.freepatentsonline.com/20070004044.html, last accessed 15 February 2021). For the quantitative analysis of energy metabolism intermediates involved in glycolysis, citrate cycle or pentose phosphate pathway, urea cycle hydrophilic interaction liquid chromatography (HILIC)-Electrospray ionization ESI-MS/MS was performed using a SCIEX 4000 QTrap^TM^ tandem mass spectrometer (Applied Biosystems/MDS Analytical Technologies) for multiple reaction monitoring (MRM). Protein was precipitated and extracted simultaneously from samples with methanol in a 96 well plate format. Ten trade secret internal standards (ratio of external to internal standard) and external calibration were used for highly accurate metabolomics quantitation, including *lactate* (Lac), *alpha-ketoglutaric acid* (alpha-KGA), *succinic acid* (Suc) and *pyruvic acid (+oxaloacetic acid)* (Pyr&OAA).

Metabolites of the energy metabolism of samples from the second stage cohorts were measured at a later timepoint according to the following procedure: After derivatization to their corresponding methoxime-trimethylsilyl (MeOx-TMS) derivatives, energy metabolites were determined by gas chromatography-mass spectrometry (GC-MS) using Agilent 7890 GC/5975 MSD (Agilent, Santa Clara, CA, USA) instruments. Pretreated samples were evaporated until completely dry and subjected to a two-step methoximation-silylation derivatization. *N*-methyl-*N*-(trimethylsilyl) trifluoroacetamide (MSTFA) was used as sialylation reagent. Split injection was performed and chromatograms were recorded in selected ion monitoring (SIM) mode. External standard calibration curves and ten internal standards were used to calculate concentrations of individual energy metabolites. Data were quantified using the appropriate mass spectrometry software (Agilent MassHunter) and imported into the Biocrates MetIDQTM software for further analysis.

As it is known that glycolysis can proceed in sampling tubes in a time depended manner, resulting in a decrease of glucose coupled with an increase of lactate [23,24], we excluded DCM samples from the analysis with H1-(Hexoses including glucose) levels less than 2-fold times standard deviation from the mean control H1-levels. H1-levels of the harmonized cohorts are shown in Supplemental Figure S1.

### 4.4. Transcriptome and Epigenome Analysis

RNAseq-analysis for polyA-enriched mRNA and measurements of CpG sites by illumina 450 k methylation array have been performed as described earlier [22]. In detail, available samples were sequenced to a median paired-end read count of 29.85 million. Unstranded paired-end raw read files were mapped with STAR [25] (v2.4.1c5) using GRCh37/hg19 and the Gencode 19 gene model (http://www.gencodegenes.org/, accessed on 17 February 2021). Only uniquely mapped reads were counted into genes using subread’s feature counts program [26] (subread version 1.4.6.p1) and mapping percentages were median 88.08. Count data were normalized by rlog normalization [27]. 

### 4.5. Statistical Analysis

To account for outliers in the raw metabolomics and methylation and RNA data, we performed outlier detection using Rosner’s Test for Outliers with from the EnvStats- (v.2.3.1) package available for R through Bioconductor (https://www.rdocumentation.org/packages/EnvStats, accessed on 17 February 2021), with k = 4 indicating the number of suspected outliers. After removal of outliers, *p*-values were calculated with help of the t.test function from the “stat_compare_means” function of the R-package ggpubr- (v.0.2.999) (https://www.rdocumentation.org/packages/ggpubr, accessed on 17 February 2021). *p*-values for individual CpG-sites have been aggregated by gene using Simes’s method for combining *p*-values (https://rdrr.io/cran/mppa/man/simes.test.html, accessed on 17 February 2021) [28].

## Figures and Tables

**Table 1:** Patient characteristics of the stage 1 cohorts.

Characteristics	DCM (*n* = 82)	Control (*n* = 51)
Male gender, n (%)	67 (82)	40 (78)
Age at visit [years] (±SD)	53.52 (±13.53)	56.22 (±8.75)
BMI [kg/m^2^] (±SD)	28.34 (±6.14)	25.64 (±2.89)
NYHA [I–IV] (±SD)	2.15 (±0.78)	0
LVEF (echo) [%](±SD)	31.05 (±13.60)	61.67 (±3.61)
eGFR [mL/min/1.73qm BSA] (±SD)	87.48 (±21.62)	90.54 (±11.08)
Smoking		
yes n (%)	18 (22)	1 (2)
no n (%)	41(20)	46(90)
ex n (%)	22 (27)	4 (8)
Diabetes	12 (15)	0 (0)

SD, standard deviation; BMI, body mass index; NYHA, New York Heart Association; LVEF, left ventricular ejection fraction; eGFR, estimated glomerular filtration rate.

**Table 2:** Patient characteristics of the stage 2 cohorts.

Characteristics	DCM (*n* = 52)	ICM (*n* = 39)	Control (*n* = 57)
Male gender, n (%)	34 (67)	32 (82)	33 (58)
Age at visit [years] (±SD)	62.12 (±12.94)	68.08 (±11.90)	62.37 (11.47)
LVEF (echo) [%](±SD)	28.03 (±11.91)	37.70 (±21.06)	58.25 (24.27)
Smoking			
yes n (%)	5 (11)	8 (21)	19 (11)
no n (%)	31 (66)	17(44)	13 (23)
ex n (%)	11 (23)	14(36)	33 (58)
Diabetes	11 (23)	13 (33)	3 (5)

SD, standard deviation; LVEF, left ventricular ejection fraction.

## Data Availability

Aggregated data of DNA methylation arrays and mRNA sequencing of the case and controls cohorts are available at https://ccb-web.cs.uni-saarland.de/cms, accessed on 17 February 2021.

## Supplementary Materials

The following are available online at https://www.mdpi.com/1422-0067/22/4/1999/s1, Figure S1: H1 metabolite levels (screening); Figure S2: Principle component analysis of metabolites (screening); Figure S3: Detection of Metabolites and associated enzyme mRNA expression (screening); Figure S4: Validation of cardiac gene expression patterns; Figure S5: Detection of Metabolites and associated enzyme methylation (screening).
