# Supplementary material for: Energy Metabolites as Biomarkers in Ischemic and Dilated Cardiomyopathy
Source: Int J Mol Sci. 2021 Feb 18;22(4):1999. doi: 10.3390/ijms22041999 (PMC7923201; doi:10.3390/ijms22041999)
Supplement: Supplementary file 1 [file ijms-22-01999-s001.zip › ijms-1094756-supplementary.pptx]

## Slide 1
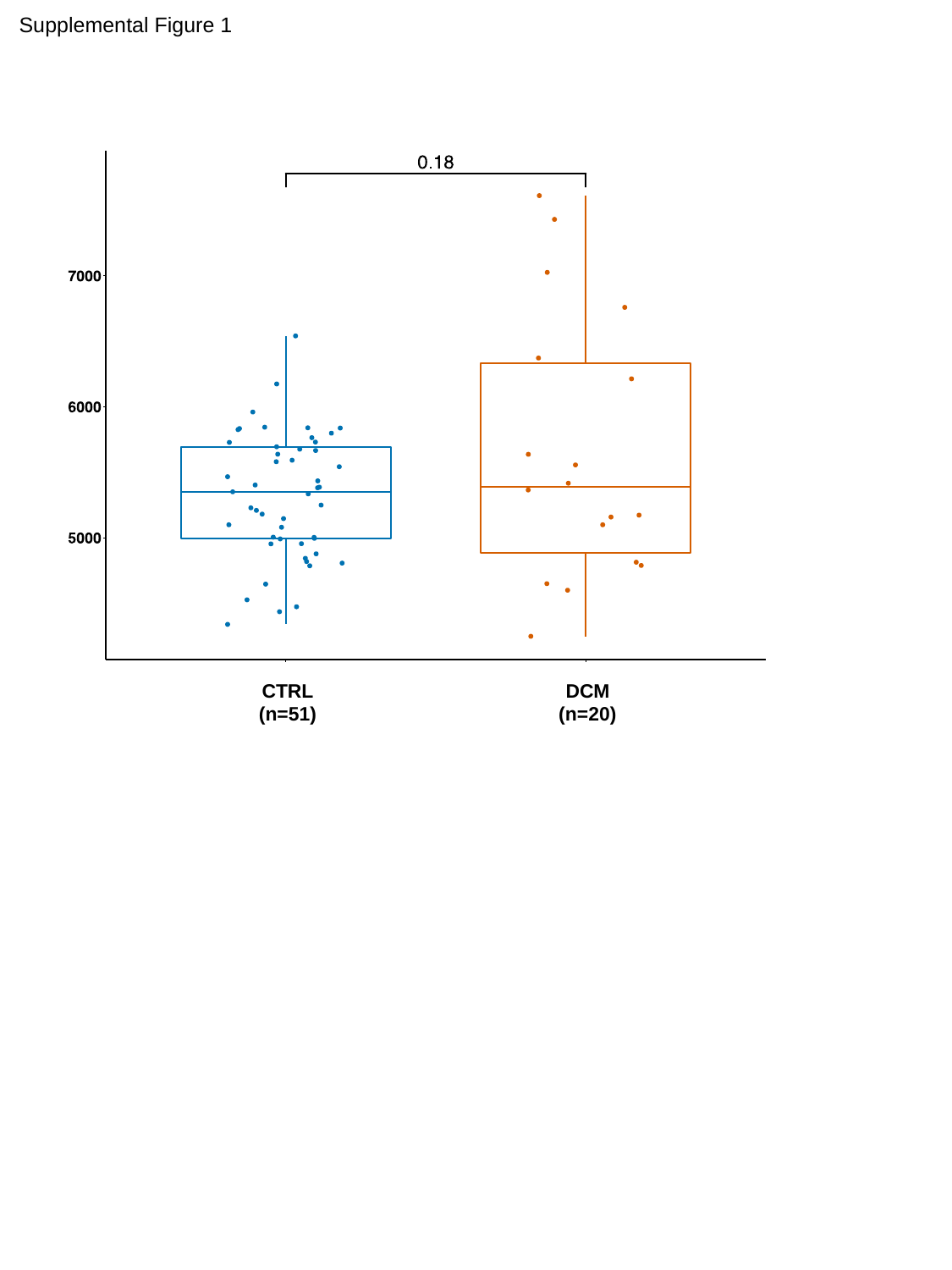

Supplemental Figure 1
CTRL
(n=51)
DCM
(n=20)

## Slide 2
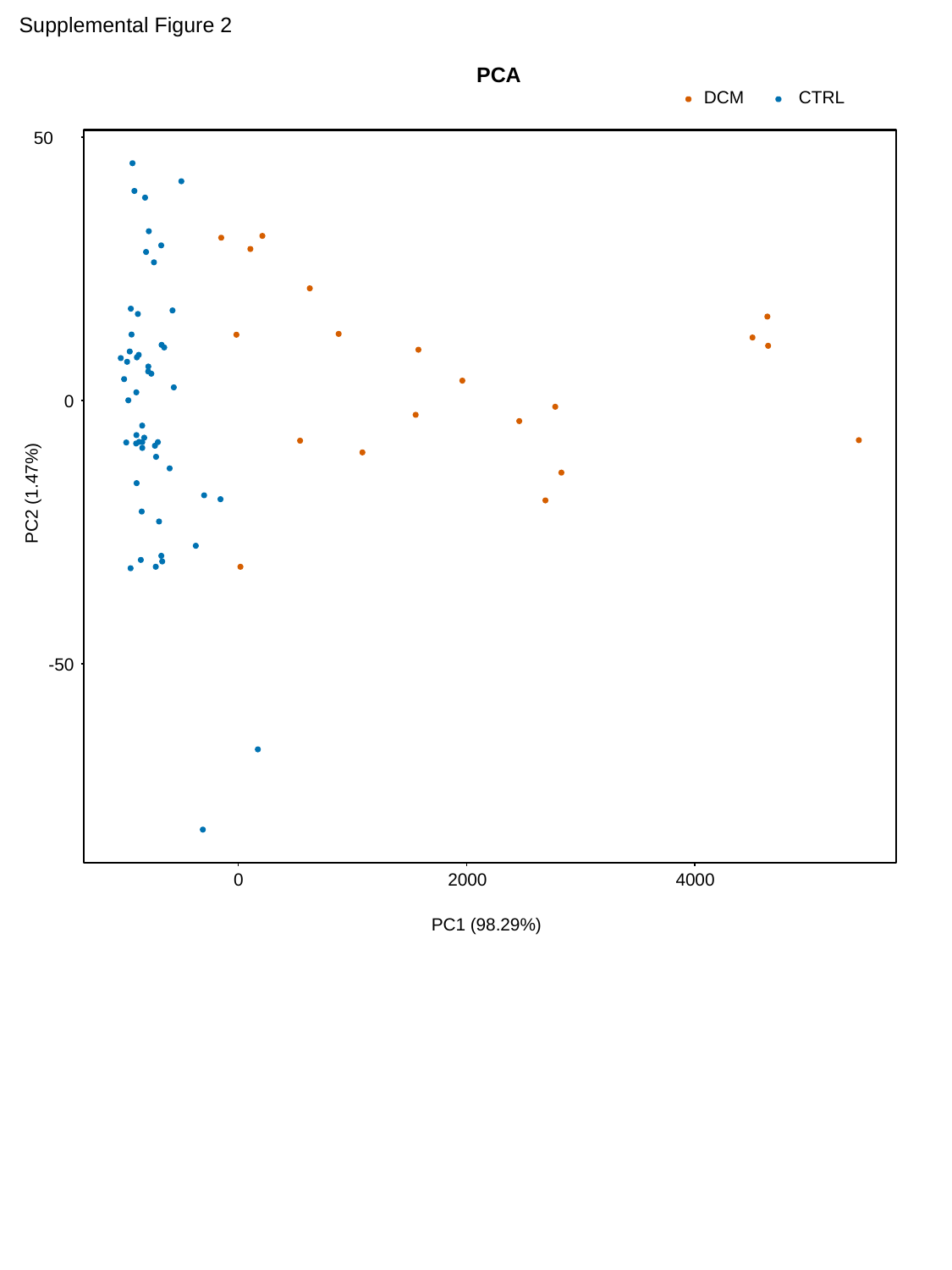

Supplemental Figure 2
PCA
DCM
CTRL
50
0
PC2 (1.47%)
-50
0
2000
4000
PC1 (98.29%)

## Slide 3
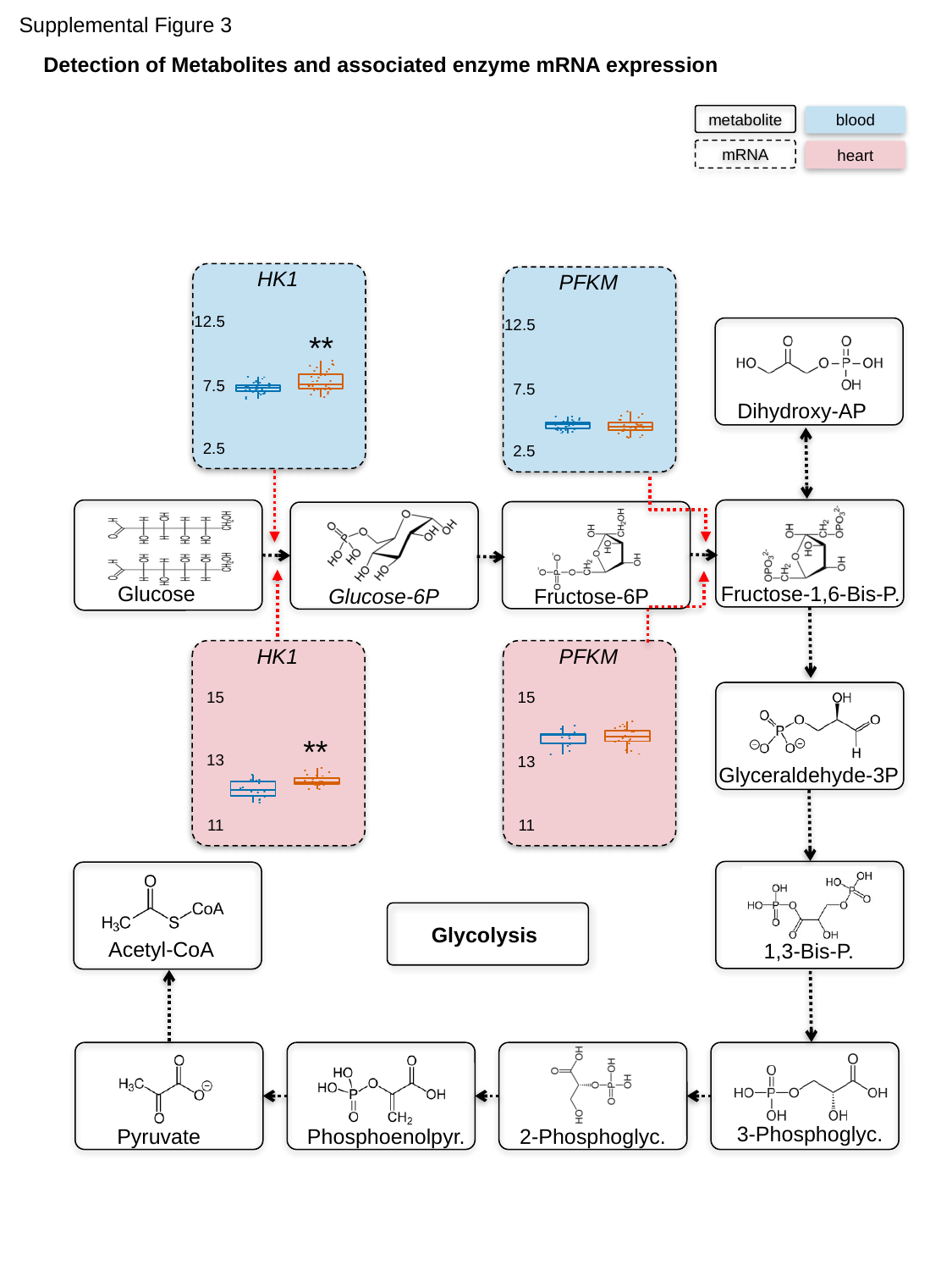

Supplemental Figure 3
Detection of Metabolites and associated enzyme mRNA expression
metabolite
blood
mRNA
heart
HK1
12.5
**
7.5
2.5
PFKM
12.5
7.5
2.5
Dihydroxy-AP
Fructose-6P
Fructose-1,6-Bis-P.
Glucose
Glucose-6P
HK1
15
**
13
11
PFKM
15
13
11
Glyceraldehyde-3P
Glycolysis
Acetyl-CoA
1,3-Bis-P.
Pyruvate
Phosphoenolpyr.
2-Phosphoglyc.
3-Phosphoglyc.

## Slide 4
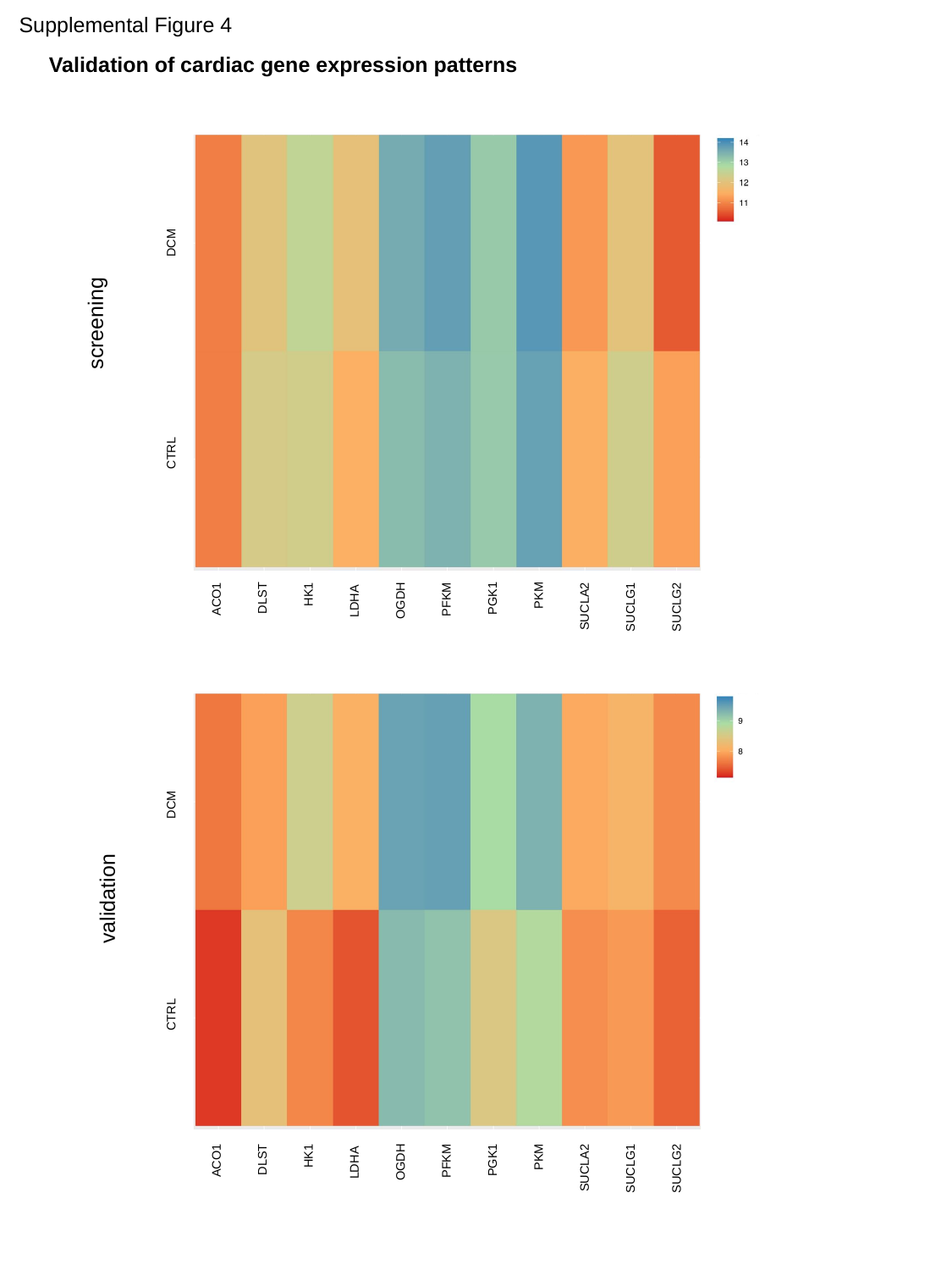

Supplemental Figure 4
Validation of cardiac gene expression patterns
DCM
screening
CTRL
PKM
HK1
LDHA
DLST
ACO1
OGDH
PFKM
PGK1
SUCLA2
SUCLG1
SUCLG2
DCM
validation
CTRL
PKM
HK1
LDHA
DLST
ACO1
OGDH
PFKM
PGK1
SUCLA2
SUCLG1
SUCLG2

## Slide 5
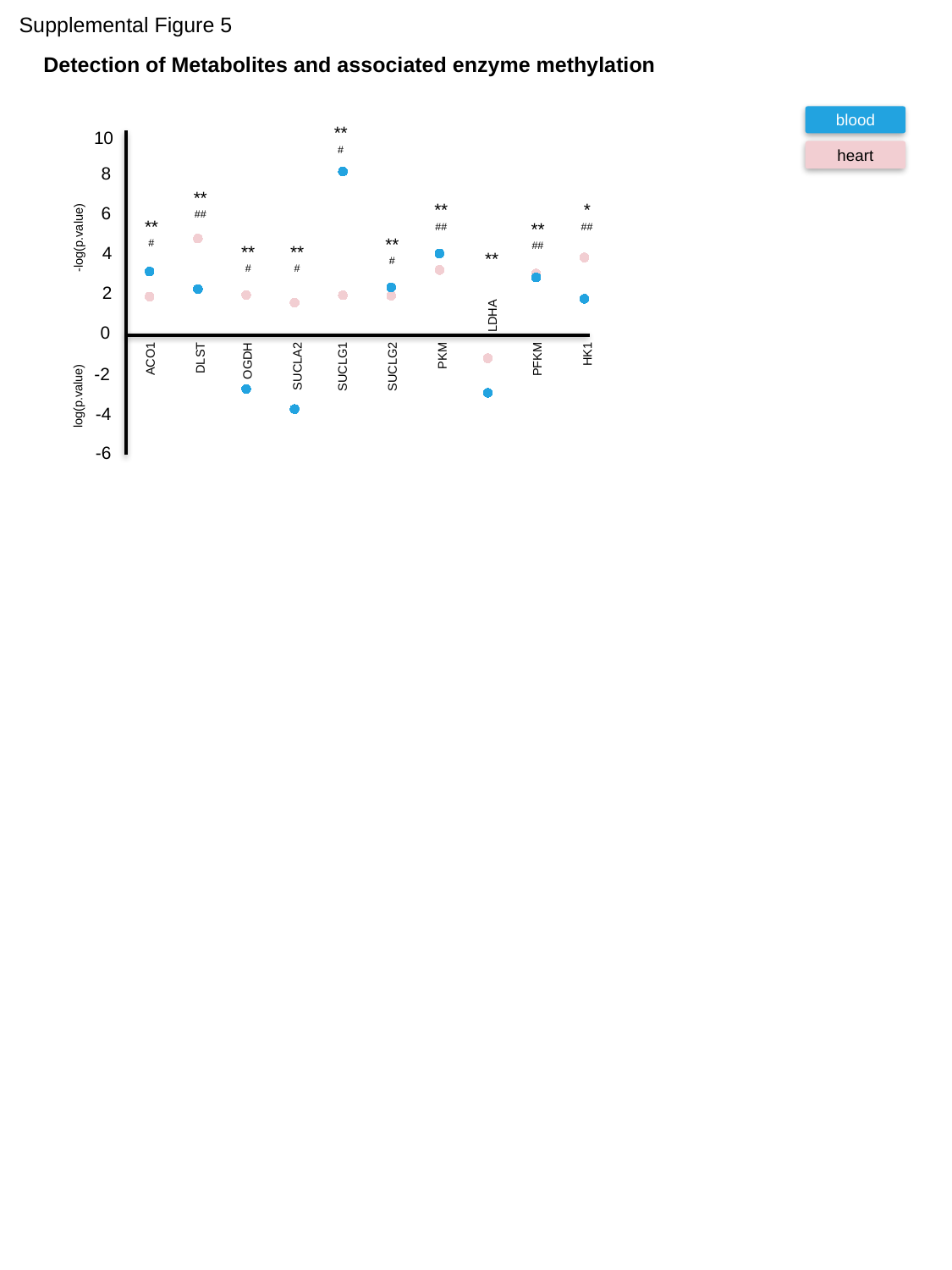

Supplemental Figure 5
Detection of Metabolites and associated enzyme methylation
blood
heart
### Chart
| Category | blood | biopsy |
|---|---|---|**
#
10
8
6
4
2
0
-2
-4
-6
**
##
**
##
*
##
**
#
**
##
-log(p.value)
**
#
**
#
**
#
**
LDHA
PKM
HK1
DLST
ACO1
OGDH
PFKM
SUCLA2
SUCLG1
SUCLG2
log(p.value)

## Slide 6
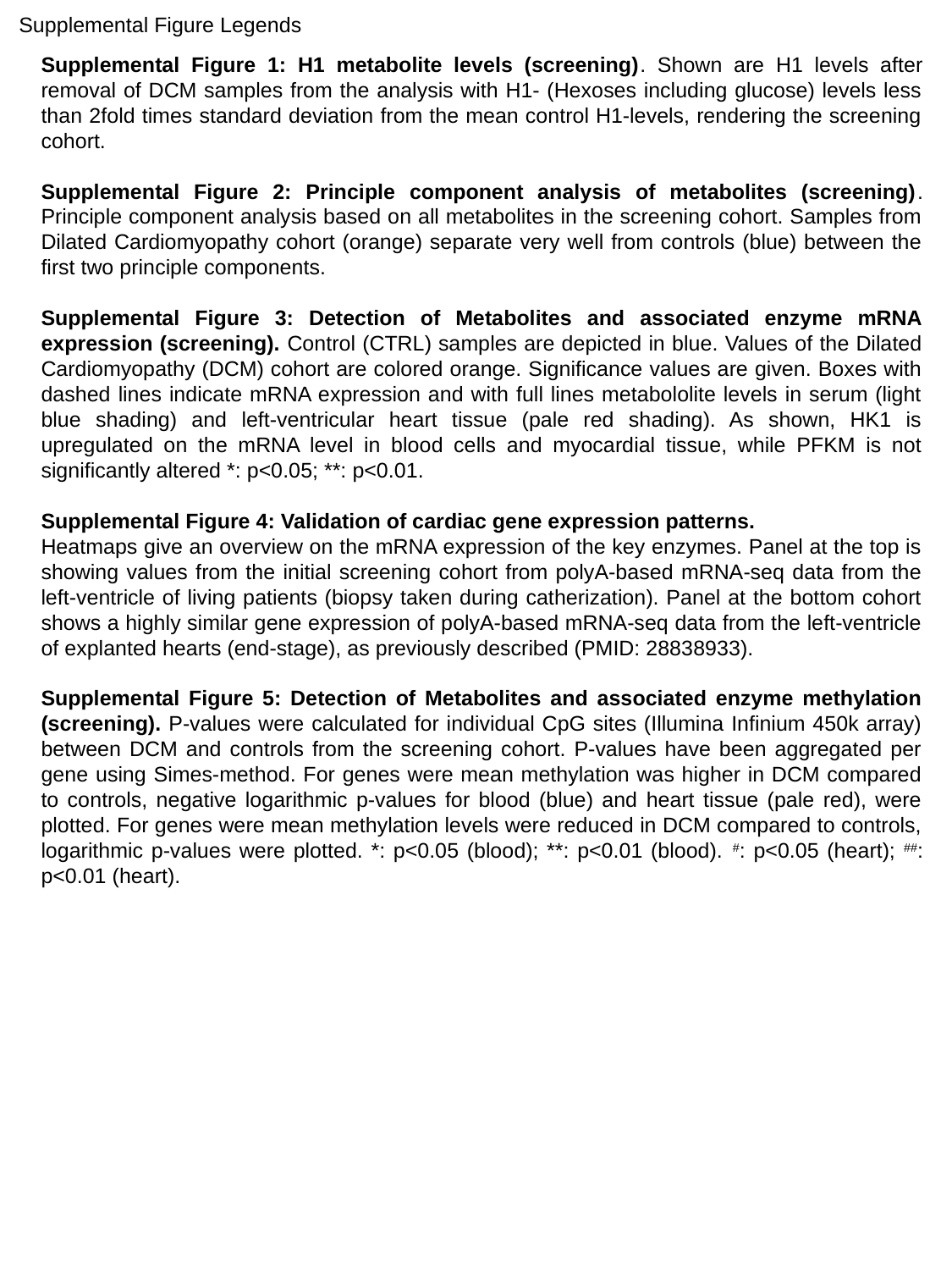

Supplemental Figure Legends
Supplemental Figure 1: H1 metabolite levels (screening). Shown are H1 levels after removal of DCM samples from the analysis with H1- (Hexoses including glucose) levels less than 2fold times standard deviation from the mean control H1-levels, rendering the screening cohort.
Supplemental Figure 2: Principle component analysis of metabolites (screening). Principle component analysis based on all metabolites in the screening cohort. Samples from Dilated Cardiomyopathy cohort (orange) separate very well from controls (blue) between the first two principle components.
Supplemental Figure 3: Detection of Metabolites and associated enzyme mRNA expression (screening). Control (CTRL) samples are depicted in blue. Values of the Dilated Cardiomyopathy (DCM) cohort are colored orange. Significance values are given. Boxes with dashed lines indicate mRNA expression and with full lines metabololite levels in serum (light blue shading) and left-ventricular heart tissue (pale red shading). As shown, HK1 is upregulated on the mRNA level in blood cells and myocardial tissue, while PFKM is not significantly altered *: p<0.05; **: p<0.01.
Supplemental Figure 4: Validation of cardiac gene expression patterns.
Heatmaps give an overview on the mRNA expression of the key enzymes. Panel at the top is showing values from the initial screening cohort from polyA-based mRNA-seq data from the left-ventricle of living patients (biopsy taken during catherization). Panel at the bottom cohort shows a highly similar gene expression of polyA-based mRNA-seq data from the left-ventricle of explanted hearts (end-stage), as previously described (PMID: 28838933).
Supplemental Figure 5: Detection of Metabolites and associated enzyme methylation (screening). P-values were calculated for individual CpG sites (Illumina Infinium 450k array) between DCM and controls from the screening cohort. P-values have been aggregated per gene using Simes-method. For genes were mean methylation was higher in DCM compared to controls, negative logarithmic p-values for blood (blue) and heart tissue (pale red), were plotted. For genes were mean methylation levels were reduced in DCM compared to controls, logarithmic p-values were plotted. *: p<0.05 (blood); **: p<0.01 (blood). #: p<0.05 (heart); ##: p<0.01 (heart).
